# First report of inter-species recombinant Gyroviruses in Chinese urban companion animals with cross-species transmission risks

**DOI:** 10.3389/fvets.2025.1619325

**Published:** 2025-06-25

**Authors:** Zhibin Zhang, Xin Xu, Dandan Li, Lunguang Yao, Jun Ji, Qingmei Xie, Yingzuo Bi

**Affiliations:** ^1^Henan Provincial Engineering Laboratory of Insects Bio-Reactor, Henan Provincial Engineering, and Technology Center of Health Products for Livestock and Poultry, Henan Provincial Engineering and Technology Center of Animal Disease Diagnosis and Integrated Control, Nanyang Normal University, Nanyang, China; ^2^College of Animal Science, South China Agricultural University, Guangzhou, China

**Keywords:** companion animals, cross-species transmission, Gyrovirus galga1, Gyrovirus homsa2, recombinant analysis, Zoonoses

## 1 Introduction

Human gyrovirus (HGyV) was first identified in skin swabs from healthy individuals and HIV-positive patients (1–3). Although HGyV is considered a member of the Gyrovirus genus, its evolutionary origin and relationship with other Gyrovirus species remain to be fully elucidated. Subsequent studies have shown that it shares more than 90% similarity with Gyrovirus galga1 (GyVg1). GyVg1 was first identified in diseased chickens in Brazil and has since been detected in a variety of hosts, including humans, ferrets, pet cats and dogs, as well as various mammals and birds in zoological settings (4–7). A study in 2012 reported that GyVg1 exhibited greater genetic variability in chickens with neurological lesions than in healthy chickens. The study further indicated that GyVg1 infection could lead to malnutrition and weight loss in chickens (8). To date, GyVg1 has been reported in multiple countries and regions, including China, Brazil, South Africa, Japan, South Korea, and Hungary (4, 9–11). Similarly, Gyrovirus homsa2 (GyVh2) was initially detected in stool samples from children with diarrhea in Tunisia, and later identified in pet cats and chickens from poultry farms (12, 13). Although GyVh2 is frequently found in diarrhea-associated samples, there is currently insufficient evidence to establish a direct association between the virus and diarrheal symptoms. The GyVg1 genome is composed of single-stranded circular DNA (ssDNA) with a total length of ~2.37–2.38 kb, and contains three open reading frames (ORFs), each encoding a distinct protein (6). VP1 contains 461 amino acids (aa), VP2 comprises 232 aa, and VP3 consists of 125 aa. Their coding sequences exhibit partial overlaps. The GyVh2 genome is ~2.3 kb in length and also encodes three proteins: the capsid protein VP1 (453 aa), VP2 (225 aa), and VP3 (111 aa) (13).

In recent years, the diversification of urban life has led to an increasingly important role of pet cats and dogs in daily life. The frequent contact between humans and pets has heightened the possibility of pets becoming potential carriers or intermediate hosts for emerging viruses. In 2019, multiple Gyroviruses, including GyVg1, were detected in pet cats in northeastern China (14). In 2022, GyVg1 was also identified in serum samples from pet dogs in other Chinese regions (15). Recent investigations into GyVg1 have shown an increasing detection rate (16). In our present study, routine disease screening of urban companion animals further confirmed the presence of GyVg1. These findings highlight the urgent need for in-depth research on the epidemiological characteristics, evolutionary trends, and potential public health implications of GyVg1 in urban pets. Therefore, studying the prevalence of GyVg1 in urban household pets is of significant importance for identifying and assessing the public health risks it may pose.

## 2 Material and methods

### 2.1 Sample processing and nucleic acid extraction

In 2024, routine disease screening of companion animals was conducted at pet hospitals in selected cities of Henan and Hubei Provinces, China. Serum samples were collected for viral nucleic acid extraction. Total viral nucleic acids were extracted using a commercial nucleic acid extraction kit (TransGen Biotechnology, Beijing, China), following the manufacturer's instructions rigorously. The eluted nucleic acid samples were stored at −80 °C in an ultra-low temperature freezer for subsequent experimental analyses.

### 2.2 Positive screening and whole-genome sequencing of recombinant strains

The extracted nucleic acid samples were initially screened using conventional PCR with GyVg1-specific primers to identify positive samples. The primers used for detection were synthesized based on sequences reported in previously published studies (7). PCR-positive samples were then subjected to segmented amplification using PrimeSTAR HS DNA polymerase (TaKaRa Bio Inc., Kusatsu, Japan) and three pairs of primers (F1: 5′-ATTTCCTAGCACTCAAAAACCCATT-3′, R1: 5′-TCTGGGCGTGCTCAATTCTGA-3′; F2: 5′-TCACAGCCAATCAGAATTGAGCACG-3′, R2: 5′-TTCTACGCGCATATCGAAATTTACC-3′; F3: 5′-TATTCCCGGAGGGGTAAATTTCGAT-3′, R3: 5′-CCCCTGTCCCCGTGATGGAATGTT-3′) specifically designed for this study. These primers covered the entire genome in three overlapping fragments: the amplified products were cloned into the pMD-18T vector (TaKaRa Bio Inc., Kusatsu, Japan) and submitted to Syn-Biotechnology Co., Ltd. (Suzhou, China) for Sanger sequencing using an ABI 3730xl DNA Analyzer (Applied Biosystems, USA). All experiments and sequencing procedures were performed in triplicate to ensure the accuracy and reliability of the results. The obtained recombinant DNA fragments were assembled using SeqMan software (DNASTAR, Lasergene^®^, Madison, Wisconsin) to generate the complete whole-genome sequences of the recombinant strains.

### 2.3 Sequence similarity and phylogenetic analysis

The newly obtained DNA sequences were pre-check through Basic Local Alignment Search Tool (BLAST; https://blast.ncbi.nlm.nih.gov/Blast.cgi). And the complete DNA genome sequences of the recombinant strains were compared with reference strains downloaded from the NCBI database using Bioinformatics Aider V1.527 to assess sequence similarity. Amino acid (aa) sequences of coding regions were also aligned to determine the genetic relationships among the strains. A total of 76 reference strains were included in this study, comprising 2 HGyV, 3 GyVh2, and 71 GyVg1 strains (Supplementary Table 1). The alignment results were visualized using the online platform Chiplot (https://www.chiplot.online/) (17). Sequence alignments were performed using the Clustal-W algorithm embedded in the Molecular Evolutionary Genetics Analysis version X (MEGA-X) software (http://www.genome.jp/tools/clustalw/) (18). Based on the optimal evolutionary model, a phylogenetic tree was constructed using the neighbor-joining (NJ) method, with 1,000 bootstrap replicates for reliability assessment. The resulting phylogenetic tree in NWK format, generated by MEGA-X, was further visualized and annotated using Chiplot (https://www.chiplot.online/).

### 2.4 Recombination prediction analysis of recombinant strains

Recombination events in the complete genome sequences of the recombinant strains were screened using Recombination Detection Program v.4.8.3 (RDP4). Under default settings, RDP4 integrates seven algorithms (MaxChi, BootScan, Chimera, 3Seq, GENECONV, SiScan, and RDP) for combined analysis to enhance the accuracy of recombination detection. Recombination signals identified by RDP4 were subsequently validated using SimPlot v.3.5.1 software. BootScan plots were generated to further confirm the recombination regions, and the results from both tools were cross validated. Finally, the recombination analysis outcomes were visualized using Origin 2022 software (OriginLab Corporation, Northampton, MA, USA).

## 3 Descriptive results

In 2024, routine disease surveillance was conducted in pet hospitals across cities in central China. A total of 213 serum samples were collected, including 101 from pet cats and 112 from pet dogs. PCR testing revealed that 12 out of 101 cats (11.88%) and 9 out of 112 dogs (8.04%) tested positive for GyVg1. These findings indicated that GyVg1 was circulating among urban companion animals and might have reached a certain level of endemicity in these populations.

Despite the detection of GyVg1-positive samples in both cats and dogs, all attempts at virus isolation were unsuccessful. To assess the possibility of recovering infectious virus, GyVg1-positive serum samples were inoculated onto Crandell–Rees feline kidney (CRFK) cells and Madin–Darby canine kidney (MDCK) cells. The cultures were maintained under standard conditions and monitored daily for cytopathic effects (CPE). After several blind passages, no apparent cytopathic changes were observed, and PCR assays of the culture supernatants failed to detect any viral DNA, suggesting an absence of viral replication in the selected cell lines. This outcome might have been attributed to low viral titers in the serum samples or the absence of appropriate cellular receptors necessary for viral entry and replication. These results imply that current *in vitro* culture systems are inadequate for GyVg1 propagation, and more effective virus isolation strategies should be explored in future studies.

**Figure 1:**
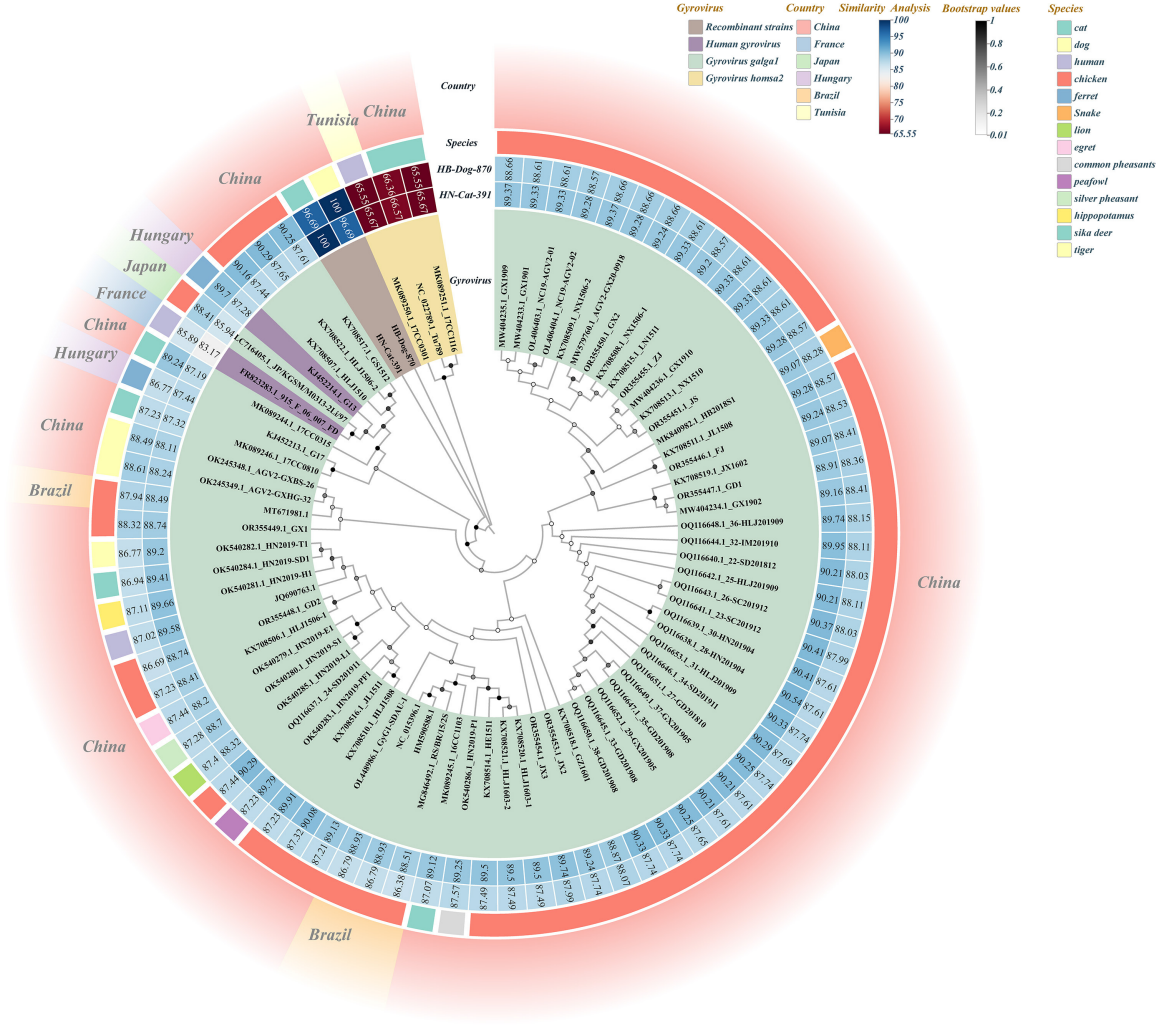
Combined diagram of the full-genome phylogenetic tree and similarity heatmap of recombinant strains and reference strains. In the Gyrovirus circular color blocks, different colors represent the recombinant strains and various other virus species. The Similarity Analysis circular color blocks indicate the similarity levels between the two recombinant strains and the reference strains, with the numbers on the blocks representing the similarity values (%). The Species circular color blocks represent the species origins of the strains used to construct the full-genome phylogenetic tree, distinguished by different colors. The outer circular color blocks use different colors to indicate the countries of origin of the strains.

**Figure 2:**
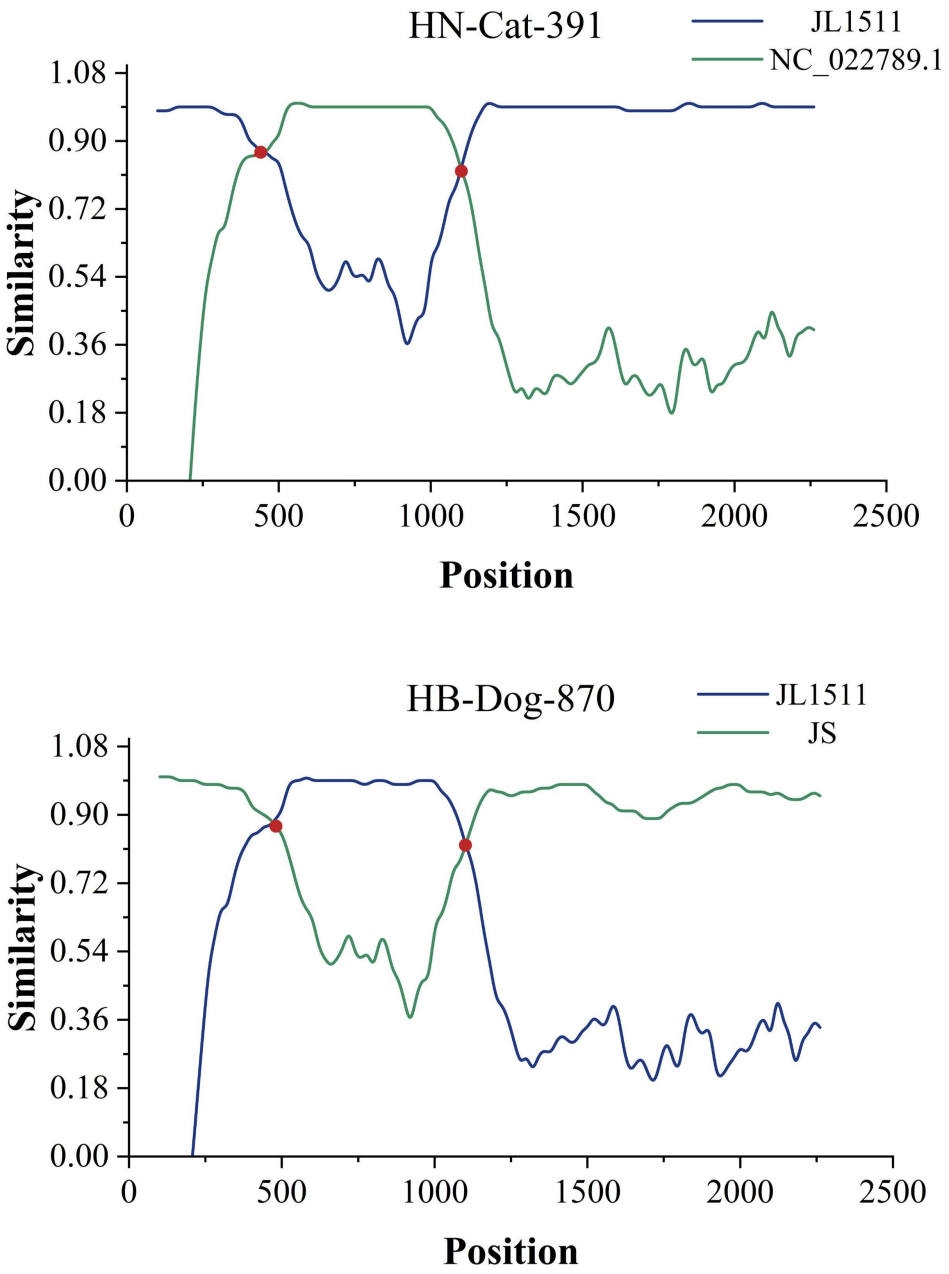
Predicted recombination events. The two recombination events were validated using SimPlot software. Recombination breakpoints are marked by the red intersections of two lines, indicating the regions where recombination likely occurred.

Based on BLAST analysis, two recombinant Gyrovirus strains, HB-Dog-870 (Accession Nos.: PV231499) and HN-Cat-391(Accession Nos.: PV231500), were identified from serum samples collected from urban pet dogs and cats. The primer binding sites were located in conserved regions of the GyVg1 genome that were also retained in the recombinant strains. As a result, the recombinant strains could still be successfully amplified using the GyVg1-based primers, despite their recombinant genomic structure. Whole-genome sequencing and recombination analysis indicated that the two strains originated from parental strains belonging to GyVg1 and GyVh2, both classified within the genus Gyrovirus characterized by broad host adaptability. Phylogenetic analysis demonstrated that HB-Dog-870 and HN-Cat-391 clustered into distinct branches, separate from previously reported Gyrovirus strains (Figure 1). During multiple sequence alignment, the recombinant strains aligned well with GyVg1 and GyVh2 reference sequences in different regions, consistent with their recombinant genomic structure. The sequence differences were primarily due to regional replacements rather than insertions or deletions, resulting in minimal gap formation. Therefore, no additional trimming or correction of alignment gaps was performed. Sequence similarity analysis showed that the recombinant strains exhibited 96.62% sequence identity, with higher similarity to GyVg1 (84.36%−90.34%) than to GyVh2 (66.92%−67.97%). The genomes of the recombinant strains were 2358 bp in length, with GyVh2 backbone containing recombination regions primarily within the overlapping VP2 and VP3 coding sequences. Recombination events in both strains were analyzed using RDP4 and SimPlot (Figure 2). The VP2 and VP3 coding regions of the recombinant strains shared identical start and stop breakpoints with GyVh2. In contrast, the VP1 coding region maintained the same overall start and stop codon positions as GyVg1, except for its initial segment which was highly similar with GyVh2. The recombination events primarily occurred in the VP2 and VP3 coding regions, which are involved in viral replication and immune evasion and may potentially influence viral fitness and pathogenicity.

The parental strains of HN-Cat-391 included NC_022789.1 (GyVh2), identified from the feces of a Tunisian child with diarrhea, while the other parental strain, KX708516.1 (GyVg1) was identified in chickens. One of the parental strains of HB-Dog-870, MK089251.1 (GyVh2), was derived from a cat, while the other parental strain, OR355454.1 (GyVg1) was identified in chickens. The recombination regions of both strains were located at specific positions in their genomes, with the recombination events of HN-Cat-391 and HB-Dog-870 further revealing the ability of Gyrovirus species to recombine across multiple hosts, suggesting the broad host adaptability of Gyrovirus species. Meanwhile, the parental strains of the two recombinant viruses were derived from chicken, cat, and human hosts, suggesting a potential risk of cross-species transmission among these hosts. The recombination event in HN-Cat-391 suggested that viral transmission among humans, chickens, and pet cats might have occurred through various pathways, including indirect transmission via human-mediated contact, environmental contamination (such as exposure to virus-contaminated surfaces or objects), or other yet unidentified routes.

The recombination event in HB-Dog-870 further supported the possibility of transmission through indirect exposure pathways rather than direct acquisition from animal-derived food sources. Based on a survey of pet owners, the companion animals in this study were exclusively fed commercial pet food, with no raw or foods of animal origin provided. Given that urban pet owners typically follow strict feeding practices, these findings support a reduced likelihood of dietary transmission. However, the frequent and close contact between companion animals and humans suggests that humans may play an important role in the cross-species spread of Gyroviruses. Such interactions increase the risk of exposure to diverse viral pathogens, and in cases of co-infection, may create conditions conducive to viral recombination and the emergence of novel variants.

Given the broad host tropism of these viruses, other transmission routes may exist in urban settings, necessitating further epidemiological studies to determine the risks associated with these emerging strains. Although no direct association with disease has been established, the identification of recombinant Gyrovirus strains in companion animals raises important concerns about their potential to cross species barriers and contribute to genetic exchange. In densely populated urban settings, close contact between humans and pets may facilitate viral evolution and increase the risk of zoonotic spillover. These findings highlight the urgent need for enhanced surveillance and further research to clarify the role of these viruses in interspecies transmission and recombination, thereby providing scientific evidence for the prevention and control of zoonotic infections.

## Data Availability

The datasets presented in this study can be found in online repositories. The names of the repository/repositories and accession number(s) can be found in the article/Supplementary material.
