# Supplementary material for: First report of inter-species recombinant Gyroviruses in Chinese urban companion animals with cross-species transmission risks
Source: Front Vet Sci. 2025 Jun 25;12:1619325. doi: 10.3389/fvets.2025.1619325 (PMC12239221; doi:10.3389/fvets.2025.1619325)
Supplement: Supplementary file 1 [file Table_1.docx]

Supplementary Material

First Report of Inter-species Recombinant Gyroviruses in Chinese Urban Companion Animals with Cross-Species Transmission Risks

**Zhibin Zhang ^1^, Xin Xu ^1^, Dandan Li ^1^, Lunguang Yao ^1^, Jun Ji ^1^,^*^, Qingmei Xie ^2^, Yingzuo Bi ^2^**

**Correspondence:** Corresponding Author: [jijun020@126.com](mailto:jijun020@126.com)

**Supplementary Table 1.** Information about sequences of reference strains used in this study

| **Accession Nos.** | **Strains** | **Host** | **Country** | **Collection date** | **Gyrovirus** |
| --- | --- | --- | --- | --- | --- |
| FR823283.1 | 915 F 06 007 FD | Human | France | 2009 | Human gyrovirus 1 |
| KJ452214.1 | G13 | Mustela putorius furo | Hungary | 2011 | Human gyrovirus 1 |
| KJ452213.1 | G17 | Mustela putorius furo | Hungary | 2011 | Gyrovirus galga1 |
| KX708522.1 | HLJ1506-2 | Gallus gallus | China | 2015 | Gyrovirus galga1 |
| KX708517.1 | GS1512 | Gallus gallus | China | 2015 | Gyrovirus galga1 |
| LC716405.1 | JP/KGSM/M0313-2Li/97 | Gallus gallus domesticus | Japan | 1997 | Gyrovirus galga1 |
| KX708507.1 | HLJ1510 | Gallus gallus | China | 2015 | Gyrovirus galga1 |
| MK089244.1 | 17CC0315 | Domestic cat | China | 2017 | Gyrovirus galga1 |
| OK245349.1 | AGV2-GXHG-32 | Dog | China | 2019 | Gyrovirus galga1 |
| OR355447.1 | GD1 | Gallus gallus | China | 2023 | Gyrovirus galga1 |
| OR355449.1 | GX1 | Gallus gallus | China | 2023 | Gyrovirus galga1 |
| KX708511.1 | JL1508 | Gallus gallus | China | 2015 | Gyrovirus galga1 |
| MW579760.1 | AGV2-GX20-0918 | Chicken | China | 2021 | Gyrovirus galga1 |
| MW404235.1 | GX1909 | Gallus gallus | China | 2019 | Gyrovirus galga1 |
| OR355450.1 | GX2 | Gallus gallus | China | 2023 | Gyrovirus galga1 |
| OR355446.1 | FJ | Gallus gallus | China | 2023 | Gyrovirus galga1 |
| OR355455.1 | ZJ | Gallus gallus | China | 2023 | Gyrovirus galga1 |
| KX708509.1 | NX1506-2 | Gallus gallus | China | 2016 | Gyrovirus galga1 |
| MW404236.1 | GX1910 | Gallus gallus | China | 2020 | Gyrovirus galga1 |
| OK245348.1 | AGV2-GXBS-26 | Dog | China | 2019 | Gyrovirus galga1 |
| KX708508.1 | NX1506-1 | Gallus gallus | China | 2015 | Gyrovirus galga1 |
| OL406403.1 | NC19-AGV2-01 | Gallus gallus | China | 2019 | Gyrovirus galga1 |
| MW404233.1 | GX1901 | Gallus gallus | China | 2019 | Gyrovirus galga1 |
| OR355451.1 | JS | Gallus gallus | China | 2023 | Gyrovirus galga1 |
| KX708515.1 | LN1511 | Gallus gallus | China | 2015 | Gyrovirus galga1 |
| OL406404.1 | NC19-AGV2-02 | Gallus gallus | China | 2019 | Gyrovirus galga1 |
| KX708513.1 | NX1510 | Gallus gallus | China | 2015 | Gyrovirus galga1 |
| MW404234.1 | GX1902 | Chicken | China | 2019 | Gyrovirus galga1 |
| KX708519.1 | JX1602 | Gallus gallus | China | 2016 | Gyrovirus galga1 |
| MK840982.1 | HB2018S1 | Snake | China | 2019 | Gyrovirus galga1 |
| MT671981.1 | - | Gallus gallus | Brazil | 2020 | Gyrovirus galga1 |
| OQ116644.1 | 32-IM201910 | Gallus gallus | China | 2019 | Gyrovirus galga1 |
| OQ116648.1 | 36-HLJ201909 | Gallus gallus | China | 2019 | Gyrovirus galga1 |
| OQ116642.1 | 25-HLJ201909 | Gallus gallus | China | 2019 | Gyrovirus galga1 |
| OQ116643.1 | 26-SC201912 | Gallus gallus | China | 2019 | Gyrovirus galga1 |
| OQ116641.1 | 23-SC201912 | Gallus gallus | China | 2019 | Gyrovirus galga1 |
| OQ116640.1 | 22-SD201812 | Gallus gallus | China | 2018 | Gyrovirus galga1 |
| OQ116645.1 | 33-GD201908 | Gallus gallus | China | 2019 | Gyrovirus galga1 |
| OQ116650.1 | 38-GD201908 | Gallus gallus | China | 2019 | Gyrovirus galga1 |
| OQ116653.1 | 31-HLJ201909 | Gallus gallus | China | 2019 | Gyrovirus galga1 |
| OQ116651.1 | 27-GD201810 | Gallus gallus | China | 2018 | Gyrovirus galga1 |
| KX708518.1 | GZ1601 | Gallus gallus | China | 2016 | Gyrovirus galga1 |
| OQ116639.1 | 30-HN201904 | Gallus gallus | China | 2019 | Gyrovirus galga1 |
| OQ116652.1 | 29-GX201905 | Gallus gallus | China | 2019 | Gyrovirus galga1 |
| OQ116646.1 | 34-SD201911 | Gallus gallus | China | 2019 | Gyrovirus galga1 |
| OQ116649.1 | 37-GX201905 | Gallus gallus | China | 2019 | Gyrovirus galga1 |
| OR355453.1 | JX2 | Gallus gallus | China | 2023 | Gyrovirus galga1 |
| OQ116647.1 | 35-GD201908 | Gallus gallus | China | 2019 | Gyrovirus galga1 |
| OQ116638.1 | 28-HN201904 | Gallus gallus | China | 2019 | Gyrovirus galga1 |
| OR355454.1 | JX3 | Gallus gallus | China | 2023 | Gyrovirus galga1 |
| OL448986.1 | GyG1-SDAU-1 | Gallus gallus | China | 2020 | Gyrovirus galga1 |
| OK540285.1 | HN2019-L1 | Lion | China | 2019 | Gyrovirus galga1 |
| OK540279.1 | HN2019-E1 | Egret | China | 2019 | Gyrovirus galga1 |
| OK540286.1 | HN2019-P1 | Common pheasants | China | 2019 | Gyrovirus galga1 |
| OQ116637.1 | 24-SD201911 | Gallus gallus | China | 2019 | Gyrovirus galga1 |
| KX708510.1 | HLJ1508 | Gallus gallus | China | 2015 | Gyrovirus galga1 |
| KX708514.1 | HE1511 | Gallus gallus | China | 2015 | Gyrovirus galga1 |
| MK089246.1 | 17CC0810 | Domestic cat | China | 2017 | Gyrovirus galga1 |
| KX708520.1 | HLJ1603-1 | Gallus gallus | China | 2016 | Gyrovirus galga1 |
| KX708516.1 | JL1511 | Gallus gallus | China | 2015 | Gyrovirus galga1 |
| KX708521.1 | HLJ1603-2 | Gallus gallus | China | 2016 | Gyrovirus galga1 |
| MK089245.1 | 16CC1103 | Domestic cat | China | 2016 | Gyrovirus galga1 |
| KX708506.1 | HLJ1506-1 | Gallus gallus | China | 2015 | Gyrovirus galga1 |
| JQ690763.1 | - | Homo sapiens | China | 2012 | Gyrovirus galga1 |
| OK540283.1 | HN2019-PF1 | Peafowl | China | 2019 | Gyrovirus galga1 |
| OK540280.1 | HN2019-S1 | Silver pheasant | China | 2019 | Gyrovirus galga1 |
| MG846492.1 | RS/BR/15/2S | Gallus gallus | Brazil | 2015 | Gyrovirus galga1 |
| OK540281.1 | HN2019-H1 | Hippopotamus | China | 2019 | Gyrovirus galga1 |
| HM590588.1 | - | Gallus gallus | Brazil | 2006 | Gyrovirus galga1 |
| NC_015396.1 | - | Gallus gallus | Brazil | 2006 | Gyrovirus galga1 |
| OK540284.1 | HN2019-SD1 | Sika deer | China | 2019 | Gyrovirus galga1 |
| OK540282.1 | HN2019-T1 | Tiger | China | 2019 | Gyrovirus galga1 |
| OR355448.1 | GD2 | Gallus gallus | China | 2023 | Gyrovirus galga1 |
| NC_022789.1 | Tu789 | Homo sapiens | Tunisia | 2003 | Gyrovirus homsa2 |
| MK089250.1 | 17CC0301 | Domestic cat | China | 2017 | Gyrovirus homsa2 |
| MK089251.1 | 17CC1116 | Domestic cat | China | 2017 | Gyrovirus homsa2 |
